# 778 Development of Medical Play Protocol for Pediatric Transparent Facial Orthosis Fabrication

**DOI:** 10.1093/jbcr/irae036.319

**Published:** 2024-04-17

**Authors:** Amanda Moore, Deborah Knight

**Affiliations:** JMS-Doctors Hospital of Augusta, Augusta, GA; Doctors Hospital- Augusta JMS Burn Center, Augusta, GA; JMS-Doctors Hospital of Augusta, Augusta, GA; Doctors Hospital- Augusta JMS Burn Center, Augusta, GA

## Abstract

**Introduction:**

Until 2021, when fabricating custom Transparent Facial Orthoses (TFO), the Occupational Therapists at our facility utilized a 3rd party to produce 3-D molds of patient faces. In order to maintain HIPAA compliance, the level of digital security needed could no longer be provided by this third party, and our therapists had to revert to a more labor-intensive process. This process, which involved casting the face with plaster strips, can feel invasive and often produces high anxiety and fear for pediatric burn survivors with facial scars. Within the Child Life domain, the benefits of medical play have been well documented and well researched. Medical play provides increased mastery, control, comfort, and understanding which all lead to improved compliance and positive coping. By incorporating medical play in the TFO fabrication process, Child Life and Occupational Therapy were able to collaborate in order to develop a protocol through which pediatric burn survivors could perform the steps of casting a doll’s face. This allowed the patient to manipulate the materials, learn the process of the intervention, and rehearse coping skills prior to receiving this intervention. Through the development of this protocol, the interdisciplinary team was able to complete 8 successful TFO fabrications within a 9 month period.

**Methods:**

Identification of need for custom fabricated TFO for pediatric burn survivors under 10 years of age, or 10 years or older with a documented history of maladaptive coping Successfully completed Child Life/Occupational Therapy medical play/TFO mold fabrication with 3 year-old patient, then continued the same process with an additional five children Interdisciplinary team assessment of TFO mold fabrication process with foundational medical play Protocol developed Protocol followed on additional two pediatric burn survivors with successful outcomes

**Results:**

Based on our assessment of five successful and one unsuccessful TFO custom mold fabrication processes, we developed a medical-play based protocol to guide the interdisciplinary team. The implementation of this protocol has reduced the anxiety and fear experienced by pediatric burn survivors with facial scars. It has also improved rapport and buy-in with caregivers and has minimized procedural time for the interdisciplinary team.

**Conclusions:**

Managing pediatric anxiety during interventions that involve close proximity to the patient’s face and restrict movement of the mouth and sometime the eyes can be challenging for the interdisciplinary pediatric burn team. By incorporating medical play as a standard of care protocol prior to these types of non-invasive but anxiety provoking procedures we have seen greater compliance and improved patient coping both in the fabrication of the TFO as well as in the ongoing wearing and usage of this method of scar management.

**Applicability of Research to Practice:**

Pediatric protocol development to improve scar management compliance

